# *PNPLA3* Genotype and Dietary Fat Modify Concentrations of Plasma and Fecal Short Chain Fatty Acids and Plasma Branched-Chain Amino Acids

**DOI:** 10.3390/nu16020261

**Published:** 2024-01-16

**Authors:** Milla-Maria Tauriainen, Susanne Csader, Maria Lankinen, Kwun Kwan Lo, Congjia Chen, Olli Lahtinen, Hani El-Nezamy, Markku Laakso, Ursula Schwab

**Affiliations:** 1 Department of Medicine, Endoscopy Unit, Kuopio University Hospital, 70029 Kuopio, Finland; 2 Institute of Public Health and Clinical Nutrition, University of Eastern Finland, 70210 Kuopio, Finlandmaria.lankinen@uef.fi (M.L.); hani.el-nezami@uef.fi (H.E.-N.); ursula.schwab@uef.fi (U.S.); 3 School of Biological Sciences, University of Hong Kong, Pokfulam, Hong Kong, China; emilylkk@hku.hk (K.K.L.); nedchen2@gmail.com (C.C.); 4 Diagnostic Imaging Centre, Department of Clinical Radiology, Kuopio University Hospital, 70029 Kuopio, Finland; olli.lahtinen@pshyvinvointialue.fi; 5 Institute of Clinical Medicine, Internal Medicine, University of Eastern Finland, 70211 Kuopio, Finland; markku.laakso@uef.fi; 6 Department of Medicine, Kuopio University Hospital, 70029 Kuopio, Finland; 7 Department of Medicine, Endocrinology and Clinical Nutrition, Kuopio University Hospital, 70029 Kuopio, Finland

**Keywords:** *PNPLA3* genotype, non-alcoholic fatty liver disease (NAFLD), dietary fat modification, diet intervention, dietary fat, fat quality, short chain fatty acids (SCFA), fecal SCFA, acetic acid, propionic acid, iso-butyric acid, butyric acid, valeric acid, branched-chain amino acids (BCAA), valine, leucine, saturated fat (SFA), monounsaturated fat (MUFA), polyunsaturated fat (PUFA), nutrigenetics

## Abstract

The GG genotype of the Patatin-like phosphatase domain-containing 3 (*PNPLA3*), dietary fat, short-chain fatty acids (SCFA) and branched-chain amino acids (BCAA) are linked with non-alcoholic fatty liver disease. We studied the impact of the quality of dietary fat on plasma (p) and fecal (f) SCFA and p-BCAA in men homozygous for the *PNPLA3* rs738409 variant (I148M). Eighty-eight randomly assigned men (age 67.8 ± 4.3 years, body mass index 27.1 ± 2.5 kg/m^2^) participated in a 12-week diet intervention. The recommended diet (RD) group followed the National and Nordic nutrition recommendations for fat intake. The average diet (AD) group followed the average fat intake in Finland. The intervention resulted in a decrease in total p-SCFAs and iso-butyric acid in the RD group (*p* = 0.041 and *p* = 0.002). Valeric acid (p-VA) increased in participants with the GG genotype regardless of the diet (RD, 3.6 ± 0.6 to 7.0 ± 0.6 µmol/g, *p* = 0.005 and AD, 3.8 ± 0.3 to 9.7 ± 8.5 µmol/g, *p* = 0.015). Also, genotype relation to p-VA was seen statistically significantly in the RD group (CC: 3.7 ± 0.4 to 4.2 ± 1.7 µmol/g and GG: 3.6 ± 0.6 to 7.0 ± 0.6 µmol/g, *p* = 0.0026 for time and *p* = 0.004 for time and genotype). P-VA, unlike any other SCFA, correlated positively with plasma gamma-glutamyl transferase (*r* = 0.240, *p* = 0.025). Total p-BCAAs concentration changed in the AD group comparing *PNPLA3* CC and GG genotypes (CC: 612 ± 184 to 532 ± 149 µmol/g and GG: 587 ± 182 to 590 ± 130 µmol/g, *p* = 0.015 for time). Valine decreased in the RD group (*p* = 0.009), and leucine decreased in the AD group (*p* = 0.043). RD decreased total fecal SCFA, acetic acid (f-AA), and butyric acid (f-BA) in those with CC genotype (*p* = 0.006, 0.013 and 0.005, respectively). Our results suggest that the *PNPLA3* genotype modifies the effect of dietary fat modification for p-VA, total f-SCFA, f-AA and f-BA, and total p-BCAA.

## 1. Introduction

The rs738409 variant (I148M) of the Patatin-like phosphatase domain-containing 3 (*PNPLA3*) gene is the most common gene associated with non-alcoholic fatty liver disease (NAFLD) [1,2], a condition contributed by genetic factors in one-third of individuals [3]. The prevalence of the G risk allele of *PNPLA3* varies from 17% to 50% in different populations [4]. In the Finnish population, the prevalence of the GG genotype is 6% [5]. Increased total energy intake and the *PNPLA3* risk genotype GG have been associated with non-alcoholic steatohepatitis (NASH) [6]. NAFLD is a common liver disease [7,8,9] associated with metabolic syndrome. Diet and genetic background affect the risk of NAFLD [3,9,10,11,12,13]. 

It has been reviewed that genetic variability modifies short-chain fatty acid (SCFA) metabolism [14], and genes and dietary fatty acids regulate fatty acid composition [15]. Western diet, including high-calorie intake and excess proportion of saturated fats (SFA), is associated with the risk of NAFLD and obesity [16,17]. SFA, but not polyunsaturated fat (PUFA), increases intrahepatic triglycerides [18,19] and obesity [20]. In Finland, the recommendation of SFA intake (<10% of total energy) is achieved by only one of 20 individuals [21]. Also, many dietary fat interventions have been reviewed in relation to NAFLD [22] but not in relation to the NAFLD risk gene *PNPLA3*. 

SCFAs are produced by the gut microbiota through saccharolytic fermentation [23] and consist of acetic acid (AA); propionic acid (PA); butyric acid (BA), including iso-butyric acid (IBA); and, to a lesser amount, valeric acid (VA). Plasma SCFAs are beneficial for health and improve intestinal barrier function [24] and are involved in energy homeostasis [25]. Plasma BA and PA serve as substrates for lipogenesis and gluconeogenesis in the liver [26]. On the other hand, high f-SCFA levels have been found to be associated with gut dysbiosis, excess adiposity, cardiometabolic risk factors [27] and NAFLD [26,28]. The branched-chain SCFA IBA is the proteolytic fermentation product of branched-chain amino acids (BCAAs) and influences glucose and lipid metabolism in adipocytes [29]. A calorie restriction diet or a low-calorie Mediterranean or vegetarian diet decreased or had no effect on f-SCFAs [30,31], but the effects of dietary fat quality modification on the SCFAs or BCAAs have not been reported yet. Interaction between the GG genotype of *PNPLA3*, essential fatty acids and carbohydrates have been reported in NAFLD [32], but the interaction of *PNPLA3* with SCFA and BCAA has not yet been published.

Plasma BCAAs, including valine (VAL), leucine (LEU) and isoleucine (ILEU), are absorbed from the diet, with meat and dairy products being the main sources [33,34]. High-protein or high-energy diets, as well as muscle catabolism, are known to increase p-BCAA concentrations [35,36]. In mice, tissue levels of BCAAs were increased by light exercise [37]. P-BCAA concentrations are higher in male, obese individuals with insulin resistance or diabetes, cardiovascular disease and subjects with NASH compared to simple liver steatosis [33,35,38,39,40,41,42], but the causative relation to these diseases is not clear [43,44,45]. In another study, obese people with NAFLD had higher p-VAL compared to lean people with NAFLD [46]. Both p-VAL and p-LEU are proposed as cardiometabolic risk factors [47]. P-ILE can prevent plasma glucose increase by stimulating glucose intake in muscle and prevent obesity and hyperinsulinemia in rats and mice [48,49].

The linkage between the *PNPLA3* gene, diet fat, NAFLD, SCFAs and BCAAs has been proposed. However, a study combining them all into one setting has not yet been published. Further, the knowledge of the effect of dietary fat modification in those with high genetic risk of NAFLD is limited. A recent review [50] suggested that having a further understanding of SCFA could allow a more personalized dietary therapy for patients with obesity, which is closely linked to NAFLD. Therefore, the primary aim of this study was to examine whether the effect of dietary fat modification on plasma and fecal SCFA and plasma BCAA differ in carriers of *PNPLA3* rs738409 *CC* or *GG* genotype. As a secondary aim, we studied the association of SCFAs and BCAAs to liver- and diabetes-associated factors and clinical characteristics.

## 2. Materials and Methods

### 2.1. Study Participants

Study participants (homozygotes for *PNPLA3* rs738409 SNP, I148M variant) were recruited from the METSIM study [51]. Inclusion criteria were the CC or GG genotypes of *PNPLA3* rs738409, body mass index (BMI) < 35 kg/m^2^, total cholesterol < 8 mmol/L, low-density lipoprotein (LDL) cholesterol < 5 mmol/L, fasting glucose < 7 mmol/L, alanine aminotransferase (ALT) < 100 U/L and age of 60–75 years. We excluded subjects with inflammatory diseases, other liver diseases besides fatty liver disease, kidney disease, unstable thyroid disease, diabetes, mental illnesses preventing the completion of the study, excess alcohol use (daily ≥ 30 g in men and ≥20 g in women) and smoking. 

**Figure 1:**
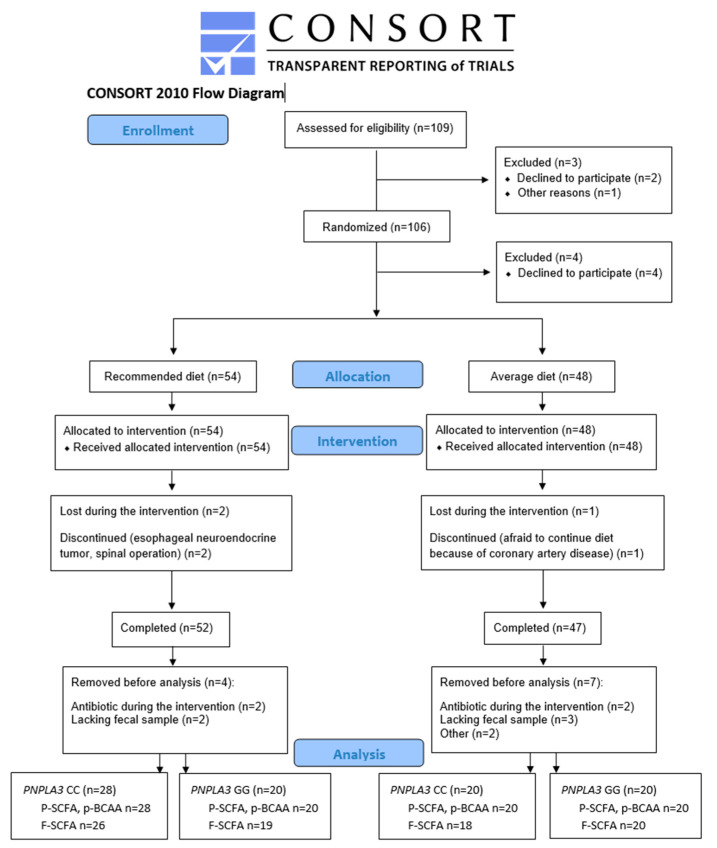
Flowchart of the LIDIGE study and analysis of short-chain fatty acids and branched-chain amino acids.

As shown in Figure 1, 109 men were identified as eligible for the study. Out of these, three were excluded before randomization (one did not arrive at the screening, and two passed the screening but chose not to participate). After randomization, four decided not to participate. Altogether, 102 men started the intervention, with 54 in the recommended diet (RD) group and 48 in the average diet (AD) group. Of those, three dropped out from the study: one in the AD group (coronary artery disease) and two in the RD group (one had an esophageal neuroendocrine tumor, and one had a spinal operation). Out of these, 99 participants completed the intervention. Four participants from the RD group and five from the AD group were removed before SCFA or BCAA analysis because they either had antibiotic treatment during the intervention or lacked fecal samples. From two participants missing the primary outcome of the study, SCFAs and BCAAs were not measured. P-SCFA and p-BCAA were analyzed from 88 and f-SCFA from 83 study participants (Figure 1). 

We included 88 men (mean age 67.8 ± 4.3 years, body mass index (BMI) 27.1 ± 2.5 kg/m^2^, waist 99.2 ± 8.8 cm) in the statistical analyses. Study participants were randomly assigned into two diet groups (RD or AD). Taking the *PNPLA3* genotype (CC or GG) into account, there were four groups. In the RD group, 28 had CC, and 20 had the GG genotype; in the AD group, 20 had CC, and 20 had the GG genotype (Figure 1 and Table 1). Background diseases or medications were not different between the study groups (Table S1). Alcohol consumption and physical exercise were kept constant during the study. BMI, waist circumference, concentrations of gamma-glutamyl transferase (GGT), fasting insulin and high-sensitive C-reactive protein (hs-CRP) were different between the study groups at baseline (*p* = 0.004, 3.6 × 10^−4^, 0.026, 0.003, 0.032, respectively) (Table 1). Covariate analyses with BMI did not change the results, and therefore, we presented data without covariation. The intake of SCFAs or BCAAs could not be calculated. 

Power calculations of the study were performed for the main outcome of the study but not for the SCFA and BCAA.

### 2.2. Genotyping for PNPLA3

The variant rs738409 (*PNPLA3*) was genotyped using the TaqMan SNP Genotyping Assay (Applied Biosystems, Foster City, CA, USA) according to their protocol. The individuals participating in this study did not know their genetic background for *PNPLA3*. The study personnel were also blinded regarding the genotype of the participants.

### 2.3. Dietary Intervention and Food Records

Dietary intervention was advised and followed by clinical nutritionists. The participants filled out four-day food records (predefined days including one weekend day) at baseline and weeks 3, 7 and 11. Records were checked by a clinical nutritionist upon their return at baseline and at weeks 4, 8 and 12.

The RD group was instructed to follow the National and Nordic nutrition recommendations [52], i.e., SFA < 10% of energy intake (E%) and unsaturated fatty acids (UFA) > 2/3 of the total fat intake. The AD group was advised to follow an average diet in Finland [21], in which the intake of SFA was aimed to be 15 E% and the proportion of UFA 50% of total fat intake.

The RD group was advised to use vegetable-oil-based spread (60–70% of fat) for bread and rapeseed oil and rapeseed-oil-based liquid products for cooking. It was recommended to use one tablespoon of oil-based salad dressing per day. Butter or butter-based spreads were not allowed. The use of juice-, yogurt- or sour cream-based dressing was not allowed. Milk and sour milk were advised to be fat-free, and yogurts were to be low-fat (fat < 1%). Cheese was advised to consist of a maximum fat of 17%, with a maximum of three to four slices per day. Low-fat (<4%) cold cuts were allowed. Fish was recommended twice a week. Two tablespoons of non-spiced nuts, seeds and almonds per day were allowed.

The AD group was advised to use butter-based spread as a spread. The salad dressing was advised to consist of, e.g., sour cream or juice, not vegetable oils. For cooking, it was advised to use butter or butter-based spreads. Milk and cultured milk were advised to consist of at least 1.5% fat, with yogurts at least 2.0%. The cheese was advised to have more than 17% fat. Fish was recommended to be eaten a maximum of once a week. Two tablespoons of nuts, seeds and almonds per week were allowed.

To provide better compliance, the key products (spreads, cooking fats and oils, and cheeses) were given to the participants for free. 

The food records were analyzed by the AivoDiet nutrient calculation software (version 2.2.0.0; Mashie FoodTech Solutions Finland Oy, Turku, Finland) based on national and international analyses and international food composition tables.

### 2.4. Laboratory Analysis

Concentrations of plasma lipids, glucose, insulin and hs-CRP were analyzed as previously described [53,54]. Liver transaminases were measured by the Eastern Finland laboratory center ISLAB.

### 2.5. Calculations for Glucose Metabolism and NAFLD Associated Scores

For glucose metabolism-associated scores, we used MATSUDA insulin sensitivity index [55], which is calculated from fasting, 30 min and 120 min (OGTT, oral glucose tolerance test) insulin and glucose concentrations as MATSUDA-ISI = 10,000/sqrt[(Ins 0 min × gluc 0 min × 18) × [(Ins 0 min + Ins 30 min + Ins 120 min)/3] × [(gluc 0 min + gluc 30 min + gluc 120 min) × 18/3)], and triglyceride-glucose (TyG) index, an indicator of insulin resistance [56], and NAFLD [57] as TyG = ln [fasting tg (mg/dL) × fasting gluc (mg/dL)]/2. 

NAFLD-associated scores included fatty liver index [58]; FLI is calculated from triglycerides (TG), BMI, GGT and waist circumference as FLI= (e ^0.953*loge (triglycerides) + 0.139*BMI + 0.718*loge (ggt) + 0.053*waist circumference − 15.745^)/(1 + e ^0.953*loge (triglycerides) + 0.139*BMI + 0.718*loge (ggt) + 0.053*waist circumference − 15.745^) × 100. Hepatic steatosis index [59] is calculated as HSI = 8 × ALT/AST + BMI(+2 if type 2 diabetes yes, +2 if female), and NAFLD liver fat score [60] is calculated as NAFLD-LFS = −2.89 + 1.18 × metabolic syndrome (Yes: 1, No: 0) + 0.45 × type 2 diabetes (Yes: 2, No: 0) + 0.15 × Ins in mU/L + 0.04 × AST in U/L − 0.94 × AST/ALT.

### 2.6. Plasma and Fecal SCFAs and Plasma BCAAs Quantification

Stool samples were collected in a plastic container with a lid by the subject at weeks 0 and 12. The sealable container was placed in a box filled with ice bags and brought to the research unit the next day. At the research unit, stool samples were directly homogenized, aliquoted and frozen at −80 °C without any detergents for further analysis. SCFAs and BCAAs were quantified using Agilent 7890B gas chromatography–mass spectrometry (GC-MS) (Agilent Technologies, Santa Clara, CA, USA), as previously described [61,62]. All the analyses were performed in blinded and random order. In brief, fecal content was mixed with 0.005 M sodium hydroxide with internal standard (10 mg/mL deuterated acetic acid), homogenized with 1.0 mm glass bead (Sigma-Aldrich, St. Louis, MO, USA) and centrifuged at 13,200× *g* for 20 min at 4 °C. Fecal supernatant or plasma was then mixed with 1-propanol/pyridine (3:2, *v*:*v*) and propyl chloroformate, incubated at 60 °C for 1 h. Hexane was then added into the derivatized samples and centrifuged at 2000× *g* for 4 min. The upper n-hexane layers were collected and analyzed with the conditions set according to Zheng et al. [61].

### 2.7. Statistical Methods

Statistical analyses were conducted by IBM SPSS Statistics for Windows, Version 27 (IBM Corp., Armonk, NY, USA), and figures were created using GraphPad Prism 5 (Boston, MA, USA). The normality of the distributions of clinical parameters, energy intake and SCFA/BCAA variables were tested using a Kolmogorov–Smirnov normality test with Lilliefors’ significance correction. Variables with skewed distribution were transformed to a base-10 logarithmic scale to achieve normal distribution. Nonparametric tests were used when a normal distribution was not achieved. A one-way analysis of variance (ANOVA) with Bonferroni’s multiple comparison test was used to test differences in baseline characteristics. Differences between the genotypes in responses to the diet, i.e., genotype–diet interaction and within the diet group’s timepoints of 0 and 12 weeks, were tested using a general linear model for repeated measures. Correlation analyses were performed as Spearman correlations at baseline.

### 2.8. Ethical Considerations

The study (Clinical Trials identifier: NCT04644887) protocol conforms to the ethical guidelines of the Declaration of Helsinki, as reflected in a prior approval by the institution’s human research committee, and has been approved by the Ethics Committee of the Northern Savo Hospital District (no:13.02.00 1408/2020). Written informed consent was obtained from each participant included in the study.

## 3. Results

### 3.1. Dietary Intervention: Recommended Diet and Average Diet

The study participants kept their diet as advised (Table 2). The intake of SFA (E%) decreased in the RD group and increased in the AD group during the intervention (*p* = 2.47 × 10^−8^ and *p* = 4.06 × 10^−8^, respectively). Additionally, the intake of both MUFA (*p* = 0.048) and PUFA increased in the RD group (*p* = 0.048 and *p* = 0.028, respectively), and PUFA decreased in the AD group (*p* = 1.20 × 10^−6^). 

Intake of omega-3 PUFA increased in the RD group (*p* = 1.8 × 10^−5^), and both omega-3 and omega-6 PUFAs decreased in the AD group (*p* = 0.004 and *p* = 1.3 × 10^−5^, respectively). Eicosapentaenoic acid (EPA) and docosahexaenoic acid (DHA) intake increased in the RD group (*p* = 2.6 × 10^−4^ and *p* = 2.2 × 10^−4^, respectively). Total fat intake (E%) decreased in the RD group (*p* = 0.032) and increased in the AD group (*p* = 0.001). Intake of fiber did not change during the study. There were no differences between the genotype groups in the adherence to the diet (based on non-significant genotype-diet interaction *p*-values, Table 2).

### 3.2. Changes in Dietary Fat Quality on Plasma SCFA and BCAA 

**Figure 2:**
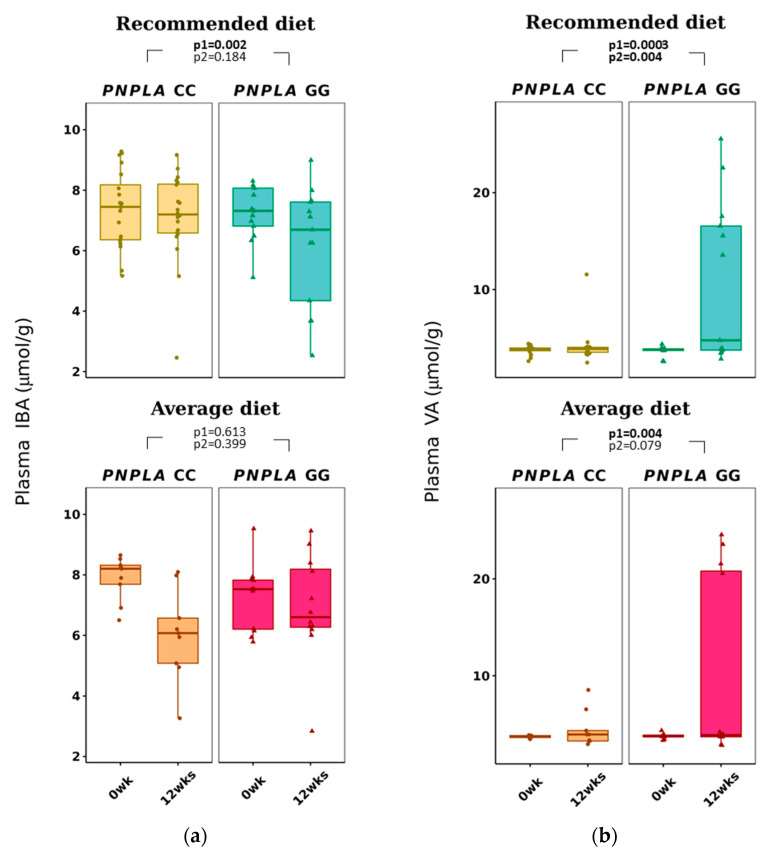
Plasma short-chain fatty acids: (**a**) iso-butyric acid (IBA) and (**b**) valeric acid (VA) at baseline (week 0) and week 12 by recommended diet and average diet and *PNPLA3* genotypes CC and GG (n = 88). Repeated generalized linear model; Values are presented as means ± SEM, dots/triangular dots present each participant; *p* < 0.05 in bold; *p*1 = time, *p*2 = time and genotype group.

Total p-SCFAs and p-IBA decreased in the RD group by time (*p* = 0.041 and *p* = 0.002, respectively) (Table 3 and Figure 2). Plasma VA increased with time in both diet groups (CC, 3.7 ± 0.4 to 4.2 ± 1.7 µmol/g; GG, 3.6 ± 0.6 to 7.0 ± 0.6 µmol/g; *p* = 0.0026 for time and *p* = 0.004 for time and genotype). 

The baseline p-IBA and p-VAL concentrations were different in comparison to the four study groups (*p* = 0.012 and *p* = 0.005, respectively, both lower in the *PNPLA3* CC genotype and AD (Table S5). At baseline, P-VAL was already lower in carriers of *PNPLA3* genotype CC (*p* = 0.014, Table S4). All p-SCFA, f-SCFA and p-BCAA changes by the diet modifications and *PNPLA3* genotypes are presented in Figure S1. 

**Figure 3:**
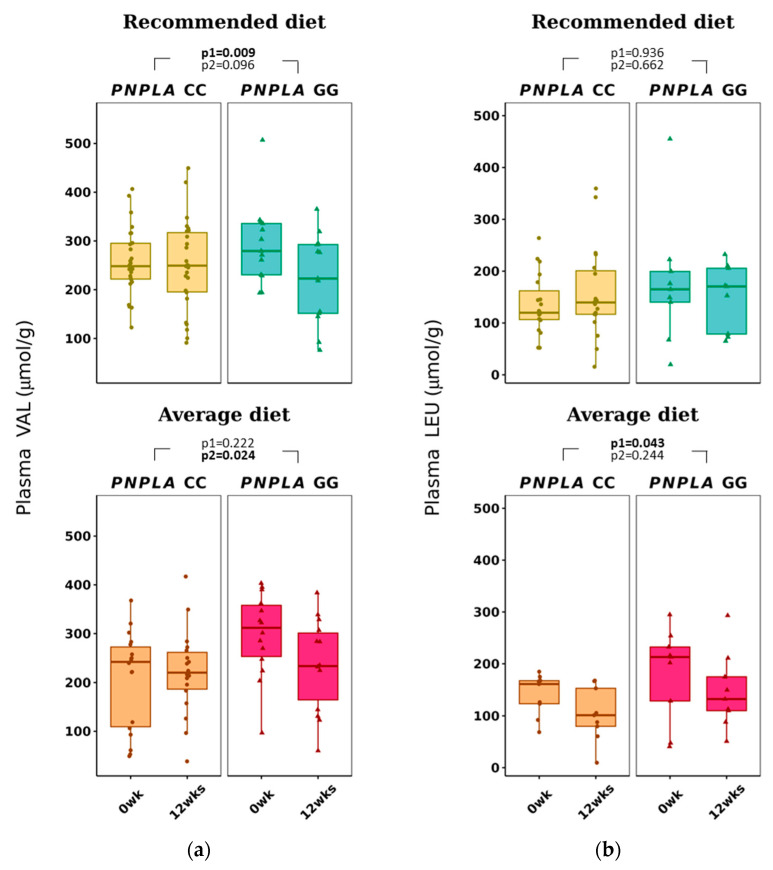
Plasma branched-chain amino acids (**a**) valine (VAL) and (**b**) leucine (LEU) at baseline (week 0) and week 12 by recommended diet and average diet and *PNPLA3* genotypes CC and GG (n = 88). Repeated generalized linear model; Values are presented as means ± SEM, dots/triangular dots present each participant; *p* < 0.05 in bold; *p*1 = time, *p*2 = time and genotype.

The average diet modified total p-BCAAs in the carriers of CC were 612 ± 184 to 532 ± 149 µmol/g (*p* = 0.015 for time) (Table 3). P-VAL decreased in the RD group (by time *p* = 0.009). Of note, VAL was already different between the *PNPLA3* genotypes *CC* and *GG* at baseline in the AD group, increased in the CC group and decreased in the GG group (*p* = 0.024, time and genotype). P-LEU decreased by time in the AD group (*p* = 0.043) (Table 3 and Figure 3). 

Plasma and fecal SCFAs did not correlate significantly with each other, and measured concentrations were in line with previous publications [63,64]. 

### 3.3. Changes in Dietary Fat Quality Modification on Fecal SCFA

**Figure 4:**
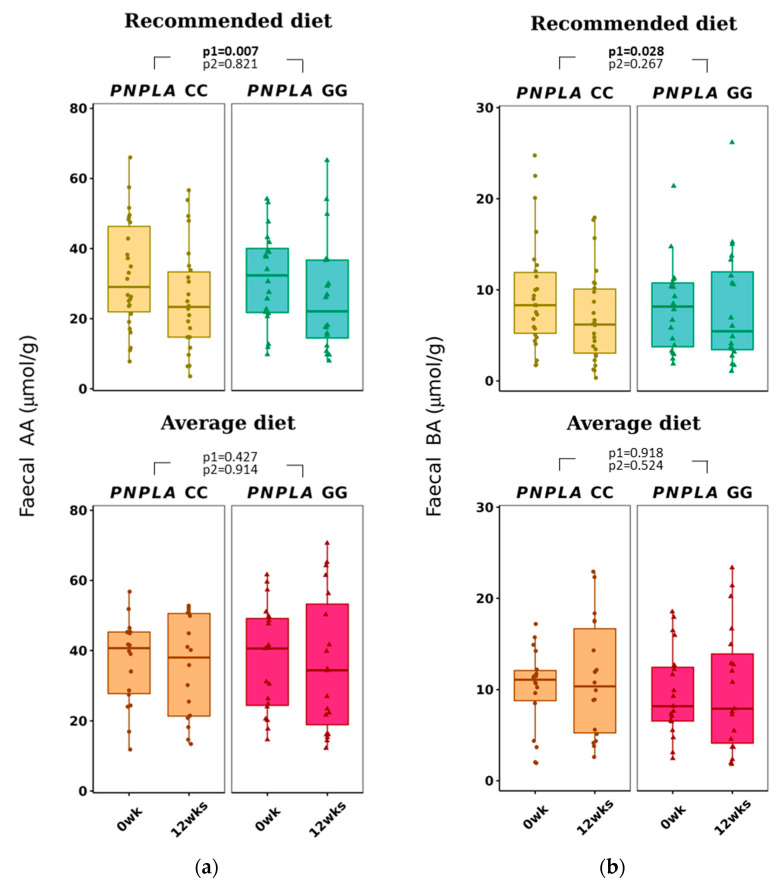
Fecal short-chain fatty acids (**a**) acetic acid (AA) and (**b**) butyric acid (BA) at baseline (week 0) and week 12 by recommended diet and average diet and *PNPLA3* genotypes CC and GG (n = 83). Repeated generalized linear model; Values are presented as means ± SEM, dots/triangular dots present each participant; *p* < 0.05 in bold; *p*1 = time, *p*2 = time and genotype.

Total f-SCFA, f-AA, and f-BA decreased in all study participants in the RD group (all *p* < 0.028 by time) (Figure 4 and Table 3). No correlations with any f-SCFAs were seen with the clinical characteristics, diabetes-related indices, or liver-related scores (Table S2A).

### 3.4. Associations of Food Intake and Plasma SCFA and BCAA and Fecal SCFA

From food intake at baseline, positive correlations were seen with energy intake and f-BA (*r* = 0.280, *p* = 0.010), with protein (E%) and total p-BCAA (*r* = 0.238 and *p* = 0.043), and carbohydrate (E%) with total f-SCFA and f-AA (*r* = 0.216, *p* = 0.0498 and *r* = 0.243 and *p* = 0.027, respectively). Also, PUFA (E%) correlated positively with total p-SCFA (*r* = 0.268, *p* = 0.016), p-AA (*r* = 0.226, *p* = 0.034) and p-BA (*r* = 0.315, *p* = 0.003), and negatively with the same SCFAs from feces (f-SCFA: *r* = −0.278, *p* = 0.011, f-AA: *r* = −0.325, *p* = 0.003 and f-BA: *r* = −0.217, *p* = 0.049). Fiber intake correlated only with p-AA (*r* = 0.211, *p* = 0.049).

### 3.5. Plasma SCFA and BCAA Associate with Lipids, Glucose Metabolism and NAFLD Associated Scores

At baseline, total plasma SCFAs and p-AA were associated with lipid metabolism by correlating positively with HDL-C concentration (*r* = 0.302 and *p* = 0.007, *r* = 0.289 and *p* = 0.006, respectively) and inversely with TG concentration (*r* = −0.307 and *p* = 0.006, *r* = −0.267 and *p* = 0.012, respectively). In addition, p-SCFA and p-AA were associated with glucose metabolism by correlating positively with MATSUDA-ISI (*r* = 0.233 and *p* = 0.038, *r* = 0.256 and *p* = 0.016, respectively) and inversely with TyG (*r* = −0.370 and *p* =0.006, *r* = −0.283, *p* = 0.008, respectively) (Table S2A).

P-VA, unlike any other SCFA, was also found to correlate positively with GGT at baseline (*r* = 0.240, *p* = 0.025) (Table S2A). 

All plasma BCAAs, valine, leucine and isoleucine had associations with glucose metabolism, seen as positive correlations with fasting insulin (*r* = 0.331 and *p* = 0.004, *r* = 0.226 and *p* = 0.036, *r* = 0.336 and *p* = 0.004, *r* = 0.284 and *p* = 0.007, respectively) and negative association with MATSUDA-ISI (*r* = −0.377 and *p* = 0.001, *r* = −0.266 and *p* = 0.013, *r* = −0.397 and *p* = 0.001, *r* = −0.309 and *p* = 0.003, respectively) and also positive correlation with NAFLD associated score LFS (*r* = 0.355 and *p* = 0.002, *r* = 0.215 and *p* = 0.046, *r* = 0.391 and *p* = 0.001, *r* = 0.329 and *p* = 0.002, respectively) (Table S2B). Also, LEU correlated positively with NAFLD-associated factors such as BMI (*r* = 0.238, *p* = 0.043), ALT (*r* = 0.295, *p* = 0.011), HIS (*r* = 0.339, *p* = 0.003) and LFS (*r* = 0.391 and *p* = 0.001). In addition, p-ILE correlated positively with TG (*r* = 0.240, *p* = 0.025); TyG, an insulin resistance index calculated from triglyceride (*r* = 0.249, *p* = 0.020); and HSI (*r* = 0.254, *p* = 0.017) (Table S2B). 

## 4. Discussion

The main result of our study is that dietary fat modification caused changes in SCFAs (p-IBA, p-VA and f-AA, f-BA) and p-BCAAs (VAL and LEU) (Figure 2 and Table 3). Previously, in humans, only the total fat (E%) intake, but not fat quality, was shown to correlate positively with acetate, butyrate and propionate [65]. On the other hand, in a study with mice, dietary fat modification resulted in changes in f-SCFA [66], but the change was probably due to a difference in fiber intake. In our study, the fiber intake was reported and kept the same, which is also a strength of this study. 

As NAFLD is a major risk factor of cardiovascular diseases and is partially caused by genetic risk factors and still missing other therapeutic approaches except weight loss, exercise and dietary changes, it is important to study whether a targeted dietary approach could provide health benefits to those in genetic risk of more progressive NAFLD. This knowledge could help in assessing the dietary advice resources better. Our study with a 12-week dietary fat modification intervention resulted in changes in plasma and fecal SCFAs and plasma BCAAs. The novelty of this study is a dietary fat quality modification with a known NAFLD-predisposing *PNPLA3* genetic background. 

In our study, RD decreased p-IBA in all study groups (Table 3), a result that we could not explain. It is worth noting that p-IBA was lower in the group with AD and PNPLA3 genotypes of CC (Table S5). An increase in IBA intake has decreased lipolysis and lipogenesis and increased insulin-stimulated glucose uptake [29] and correlates negatively with GGT [67]. In contrast, we did not see associations of p-IBA to glucose or lipid metabolism, nor NAFLD-related scores or liver transaminases (Table S2A). 

Interestingly, p-VA increased in all study groups regardless of the diet group, and the increase was greater in those with the *PNPLA3 GG* genotype (Table 3). Fecal VA has previously been associated with reduced hypercholesterolemia and lipid accumulation, total cholesterol, TG, free fatty acids or LDL-C in the liver and serum in mice [68], and lower levels of f-VA have been associated with lower GGT [67]. Since increased f-VA contributes to health benefit changes and p-VA is increased by AD with participants of the *PNPLA3* GG genotype, we suggest that *PNPLA3* and dietary fat changes would modify the absorption of VA from the gut into blood circulation. 

In contrast to previous publications [67,68], there were no correlations of p- or f-VA with lipid or glucose metabolism (Table S2A) in our study. Also, p-VA or f-VA did not correlate with other plasma or fecal SCFA or energy intake at baseline. Bearing in mind that p-VA was positively associated with GGT (Table S2A), there could be a pathway of p-VA causing more progressive NAFLD in those with the *PNPLA3* GG genotype. Unfortunately, p-VA is often lacking in publications about SCFAs, as in this recent review [30], making it even more important to report our findings. 

In our study, the fecal and plasma SCFAs did not correlate with their counterparts, unlike, i.e., in a human adult study (n = 441) from Columbia [27] or in a small pilot study (n = 10) by Vogt and Wolever [69]. Also, Deng et al. [70] reported recently that counterparts of plasma and fecal SCFAs have different associations with gut microbiota and diabetes in a cohort of 1007 middle-aged and elderly adults. Our finding was that f-AA, but not p-AA, decreased in all with RD (Table 3, Figure 4), but opposite to p-AA, which was found to associate with concentrations of HDL-C and TG, and diabetes indices (Table S2A); f-AA did not correlate with clinical characteristics, diabetes, or liver-related indices. This discrepancy of plasma and fecal SCFA values could be partly explained by the fact that only 5% of the SCFAs absorbed into circulation are excreted into feces [71] and also by the knowledge of big inter-individual variation in the concentration of f-SCFAs [72]. 

Recommended diet decreased f-BA in all subjects regardless of the *PNPLA3* genotype (Table 3), and f-BA did not correlate with clinical characteristics, diabetes indices (our study subjects were non-diabetic) or NAFLD-related scores (Table S2A). Our result did not support previous findings that reported higher fecal BA in obese people; higher total energy intake; higher fasting concentrations of TG, insulin, and hs-CRP [27]; and lower f-BA in those with type 2 diabetes [65]. Fecal BA, unlike p-BA, has been reported to be associated with gut microbiota and type 2 diabetes [70]. 

All plasma BCAAs, p-VAL, p-LEU and p-ILE were associated positively with worsening of insulin resistance (Table S2B), which is in line with previous publications [33,35,38,39,40,41,42]. In our study, AD decreased p-LEU concentrations (Table 3 and Figure 3) and was associated positively with NAFLD predisposing factors BMI, ALT, HSI and LFS (Table S2B). In addition, p-ILE correlated positively with TG and HSI (Table S2B). 

The intake and plasma concentrations of BCAAs have only a weak association [73,74,75]; therefore, the lack of BCAA intake data was not crucial in our study. In a study of adolescents, BCAAs in plasma, but not BCAA intake, were associated with obesity and insulin resistance [76]. Previously, BCAAs (mostly p-LEU [77]) have been shown to activate the mammalian target of the rapamycin (*mTOR*) gene [78], and, by using phosphoinositide-3-kinase-protein kinase B (PI3K-Akt) signaling pathway, BCAAs could modify the lipid and glucose metabolisms [79]. As BCAAs are also synthesized for circulation by the gut microbiome [80], future analyses could aim to study the relationship between BCAAs, gut microbiome, lipid and glucose metabolism and NAFLD. 

Plasma VAL was higher at baseline in participants with the *PNPLA3* genotype of GG compared to CC (Table S4), a result that has not been published before. Interestingly, both RD and AD reduced the concentrations of p-VAL with the *PNPLA3* genotype of GG (Table S3). Even though the response to AD in p-VAL was significantly different between *PNPLA3* genotypes of CC and GG, this could be due to the difference in the p-VAL concentrations at baseline (Tables S3–S5) and might not be a relevant result. In our study, p-VAL did not correlate with BMI or waist circumference, even though it has been reported to be lower in lean people with NAFLD compared to obese people with NAFLD [46]. Total BCAA, VAL, LEU and ILE correlated positively with insulin and negatively with diabetes-associated index MATSUDA (Table S2B), which is in line with higher p-BCAAs being related to worsening of glucose metabolism [41]. 

In our study, p-VAL decreased in both diets in the carriers of the GG genotype of the *PNPLA3* gene, and p-LEU decreased by AD (Table S3 and Table 3). This could suggest that *PNPLA3* modifies the concentration of p-BCAAs. In a Chinese 1-year exercise intervention, the increase in p-BCAAs improved liver fat content in obese individuals [81]. On the contrary, Merz et al. [82] noticed (in a cross-sectional study of 312 healthy adults) that an unhealthy diet was associated with higher p-BCAAs. Because some gut microbes can produce not only SCFAs but also BCAAs [80], and a high-fat diet is suggested to modify gut microbiota and increase gut permeability [83,84], the results of our study could derive from gut microbiota changes. Thus, possibly, the *PNPLA3* gene could also modify the gut microbiota in hand with the dietary fat modification and cause our differing results of SCFAs and BCAAs according to the CC or GG genotype of the *PNPLA3* gene.

The strengths of this study are the targeted recruitment of participants based on their rs738409 variant (I148M) of the *PNPLA3* gene homozygous status and the equally distributed sample size in four study groups (n = 28, 20, 20 and 20). Additionally, the participants were motivated; thus, the number of dropouts was low. The intervention was carefully guided, and the quality of the diet was well monitored. The physical activity of the participants was registered by questionnaires and remained unchanged during the intervention. In addition, the clinical parameters were not seen to change during the intervention (Table S6) besides lipids, which the diet intervention aimed to affect. 

Some study limitations need to be highlighted. The intake of carbohydrates was unintentionally changed in the AD group (increased in CC and decreased in GG genotype, Table 2). Herein, as SCFAs are derived from carbohydrates, this could cause false positive findings in genotype comparisons of SCFAs. Of note, p-IBA and p-VAL baseline concentrations were lower with the CC genotype of *PNPLA3* and AD (Table S5), and p-VAL was lower in CC than the GG genotype of PNPLA3 (Table S4). This could cause false positive findings for p-IBA and p-VAL during the intervention. 

For analyzing data, we made several comparisons between the diet groups and *PNPLA3* genotypes, and by using the p-value threshold of 0.05 instead of correcting it by the number of variables, it is possible that we had some false positive findings. Additionally, the power calculation was not based on these endpoints, so it might be that we do not have enough power for these analyses.

Combining the results of this article with liver imagining and gut microbiota analysis, as in previous publications [14,27,28,85], could help understand the effects of dietary fat modification and carriage of CC or GG genotype of *PNPLA3* gene on SCFAs and BCAAs and their possible effect on NAFLD. Our study does not provide a mechanistic answer on how the *PNPLA3* gene, together with changes in dietary fat, modifies SCFA or BCAA. The modification of diet fat could affect the gut microbiota, causing changes in gut permeability and the ability to both produce and absorb SCFAs and BCAAs. 

## 5. Conclusions

In conclusion, dietary fat modification influenced p-SCFA, f-SCFA and p-BCAA concentrations. Additionally, the *PNPLA3* gene modified the response to dietary fat modification for p-VA and total p-BCAA. Also, total p-SCFA and p-AA were associated with plasma lipids and diabetes indices, and p-BCAA was associated with diabetes indices and liver steatosis scores. Further studies addressing this issue are warranted.

## Figures and Tables

**Table 1:** Baseline characteristics of the participants (n = 88).

	CC Genotype of *PNPLA3*		GG Genotype of *PNPLA3*		
	RD n = 28	AD n = 20	RD n = 20	AD n = 20	*p* Value
Age (years)	68.9 ± 4.5	66.2 ± 3.7	68.4 ± 4.5	67.2 ± 4.2	0.151
BMI (kg/m^2^)	27.8 ± 2.5	28.1 ± 2.4	25.8 ± 2.0	26.4 ± 2.3	0.004 *^,2,3^
Waist (cm)	101.5 ± 8.7	104.4 ± 8.0	94.6 ± 7.6	95.8 ± 7.5	0.00036 *^,2,3^
GGT (U/L)	32.8 ± 19.3	32.8 ± 21.1	20.1 ± 5.6	26.1 ± 9.9	0.026 *^,2,4^
ALT (U/L)	26.3 ± 13.3	27.7 ± 9.1	23.5 ± 12.9	27.7 ± 10.6	0.633
AST (U/L)	28.2 ± 7.4	27.6 ± 5.0	27.0 ± 6.9	27.0 ± 7.5	0.902
Albumin (g/dL)	38.3 ± 2.8	38.9 ± 2.2	38.0 ± 2.4	38.5 ± 3.2	0.763
Total cholesterol (mmol/L)	4.21 ± 0.97	4.66 ± 0.96	4.51 ± 0.80	4.58 ± 1.01	0.355
HDL cholesterol (mmol/L)	1.47 ± 0.54	1.29 ± 0.25	1.49 ± 0.37	1.41 ± 0.37	0.230
LDL cholesterol (mmol/L)	2.53 ± 0.77	3.04 ± 0.86	2.84 ± 0.78	2.93 ± 0.89	0.167
Triglycerides (mmol/L)	0.97 ± 0.37	1.28 ± 0.42	1.21 ± 0.79	1.12 ± 0.49	0.193
Fasting glucose (mmol/L)	5.71 ± 0.45	5.78 ± 0.35	5.58 ± 0.35	5.83 ± 0.41	0.229
120 min glucose (mmol/L)	6.23 ± 1.46	6.31 ± 1.67	5.81 ± 1.46	5.96 ± 1.16	0.648
Fasting insulin (mU/L)	9.1 ± 5.4	14.0 ± 7.6	7.4 ± 3.5	9.1 ± 5.4	0.0031 *^,1,4^
120 min insulin (mU/L)	63.2 ± 58.6	59.8 ± 40.6	40.9 ± 27.2	52.1 ± 46.3	0.387
Hs-CRP (mg/L)	1.01 ± 1.04	1.65 ± 1.45	0.71 ± 0.31	1.02 ± 0.87	0.032 *

RD, recommended diet; AD, average diet; BMI, body mass index; GGT, gamma-glutamyl transferase; ALT, alanine aminotransferase; AST, aspartate aminotransferase; HDL, high-density lipoprotein; LDL, low-density lipoprotein; Hs-CRP, high-sensitive C-reactive protein; Mean ± SD; * *p* < 0.05, ^1^ between all the study groups, one-way ANOVA; ^2^ between the RD *CC* and *GG*; ^3^ between the AD *CC* and *GG*; ^4^ between the *CC* RD and AD.

**Table 2:** Energy intake at baseline (week 0) and during the intervention (average of weeks 3, 7 and 11) (n = 88).

*PNPLA3* Genotype	Recommended Diet				*p*1	*p*2	Average Diet				*p*1	*p*2
	CC		GG				CC		GG			
Total, n	28		20				20		20			
Study Time	0	Inter.	0	Inter.			0	Inter.	0	Inter.		
Energy (kcal/day)	2220 ± 425	2277 ± 444	2195 ± 402	2139 ± 423	0.893	0.209	2429 ± 406	2580 ± 454	2342 ± 510	2284 ± 399	0.683	0.057
Protein (E%)	16.4 ± 2.8	17.1 ± 2.3	17.5 ± 2.5	17.4 ± 1.6	0.313	0.209	16.3 ± 2.7	15.6 ± 1.8	16.8 ± 3.1	16.0 ± 2.3	0.039 *	0.914
Carbohydrate (E%)	40.8 ± 5.5	41.3 ± 5.6	42.0 ± 6.1	43.0 ± 5.5	0.218	0.880	43.2 ± 4.8	40.9 ± 5.0	43.6 ± 4.8	40.7 ± 4.7	0.001 *	0.327
Fat (E%)	38.3 ± 4.5	36.5 ± 3.5	36.2 ± 5.5	35.1 ± 4.8	0.032 *	0.606	36.8 ± 4.8	38.9 ± 4.4	35.0 ± 5.1	38.0 ± 4.7	0.001 *	0.327
SFA (E%)	13.2 ± 2.6	10.9 ± 1.9	12.3 ± 2.6	10.3 ± 1.8	2.47^−8^ *	0.616	13.0 ± 1.5	16.6 ± 2.5	12.3 ± 3.0	15.7 ± 2.0	4.06^−8^ *	0.778
MUFA (E%)	11.1 ± 2.4	14.6 ± 1.8	13.5 ± 2.6	14.2 ± 2.4	0.048 *	0.750	13.3 ± 1.9	12.9 ± 1.4	12.2 ± 2.1	12.8 ± 2.1	0.673	0.175
PUFA (E%)	7.3 ± 1.7	7.7 ± 1.3	7.1 ± 2.0	7.5 ± 1.4	0.028 *	0.798	7.2 ± 2.2	5.4 ± 0.8	6.9 ± 2.2	5.3 ± 0.7	1.20^−6^ *	0.720
Omega 3 (E%)	1.84 ± 0.61	2.16 ± 0.44	1.83 ± 0.73	2.18 ± 0.49	0.000018 *	0.671	1.78 ± 0.48	1.40 ± 0.23	1.7 ± 0.7	1.4 ± 0.2	0.004 *	0.241
Omega 6 (E%)	5.0 ± 1.2	5.3 ± 1.0	5.1 ± 1.8	5.0 ± 1.0	0.210	0.522	5.0 ± 1.5	3.9 ± 0.5	4.8 ± 1.4	3.7 ± 0.5	0.000013 *	0.980
EPA (E%)	0.056 ± 0.069	0.070 ± 0.045	0.064 ± 0.084	0.083 ± 0.056	0.00026 *	0.996	0.047 ± 0.060	0.023 ± 0.021	0.068 ± 0.071	0.035 ± 0.030	0.178	0.830
DHA (E%)	0.146 ± 0.203	0.178 ± 0.137	0.170 ± 0.245	0.227 ± 0.162	0.00022 *	0.957	0.127 ± 0.167	0.587 ± 0.542	0.175 ± 0.202	0.100 ± 0.793	0.324	0.886
Fiber (g/day)	29.3 ± 12.3	30.3 ± 10.2	31.8 ± 11.7	31.0 ± 10.8	0.709	0.224	31.2 ± 8.3	30.1 ± 9.3	29.6 ± 8.0	27.5 ± 7.2	0.064	0.717

AD, average diet; RD, recommended diet; E%/day, percent of the total energy intake of the day; SFA, saturated fat; MUFA, monounsaturated fat; PUFA, polyunsaturated fat; EPA, eicosapentaenoic fatty acid; DHA, docosahexaenoic acid; mean ± SD; repeated generalized linear model; * *p* < 0.05; *p*1 = time, *p*2 = time and genotype group.

**Table 3:** Plasma SCFA and BCAA and fecal SCFA at baseline (week 0) and after the intervention (week 12).

	Recommended Diet				*p*1	*p*2	Average Diet				*p*1	*p*2
	CC		GG				CC		GG			
Week	0	12	0	12			0	12	0	12		
**Plasma SCFA** (µmol/g)	**n = 28**		**n = 20**				**n = 20**		**n = 20**			
Total SCFA	245 ± 130	227 ± 54	232 ± 68	209 ± 85	0.041 *	0.315	228 ± 48	235 ± 78	219 ± 63	208 ± 61	0.888	0.934
Acetic acid (AA)	179 ± 121	145 ± 31	162 ± 63	143 ± 41	0.120	0.879	155 ± 33	167 ± 59	161 ± 60	142 ± 61	0.420	0.211
Propionic acid (PA)	48 ± 27	64 ± 52	43 ± 31	42 ± 48	0.700	0.354	52 ± 31	50 ± 44	36 ± 27	29 ± 34	0.077	0.983
Iso-butyric acid (IBA)	7.3 ± 1.4	6.6 ± 2.0	7.3 ± 1.3	5.7 ± 2.3	0.002 *	0.184	5.6 ±3.0	5.4 ± 2.5	7.3 ± 1.6	5.9 ± 2.5	0.613	0.399
Butyric acid (BA)	7.4 ± 1.0	7.3 ± 1.0	7.3 ± 1.3	5.7 ± 2.3	0.160	0.635	7.0 ± 0.7	7.2 ± 1.0	7.3 ± 0.7	6.9 ± 0.7	0.411	0.065
Valeric acid (VA)	3.7 ± 0.4	4.2 ± 1.7	3.6 ± 0.6	7.0 ± 0.6	0.0003 *	0.004 *	3.5 ± 0.6	4.3 ± 2.1	3.8 ± 0.3	9.7 ± 8.5	0.004 *	0.079
**Plasma BCAA** (µmol/g)												
Total BCAA	572 ± 128	581 ± 182	599 ± 202	634 ± 154	0.688	0.280	612 ± 184	532 ± 149	587 ± 182	590 ± 130	0.015 *	0.376
Valine (VAL)	258 ± 67	252 ± 90	266 ± 92	212 ± 95	0.009 *	0.096	196 ± 100	214 ± 89	290 ± 75	211 ± 108	0.222	0.024 *
Leucine (LEU)	136 ± 55	145 ± 85	141 ± 96	142 ± 59	0.936	0.662	141 ± 49	124 ± 71	162 ± 75	148 ± 61	0.043 *	0.244
Isoleucine (ILE)	160 ± 23	153 ± 33	169 ± 65	157 ± 90	0.133	0.657	164 ±112	155 ± 31	167 ± 26	139 ± 37	0.238	0.081
**Fecal SCFA** (µmol/g)	**n = 26**		**n = 20**				**n = 18**		**n = 19**			
Total SCFA	52 ± 25	40 ± 24	48 ± 21	42 ± 26	0.010 *	0.461	59 ± 22	57 ± 27	58 ± 25	60 ± 35	0.659	0.964
Acetic acid (AA)	32 ± 15	26 ± 15	32 ± 13	26 ± 16	0.007 *	0.821	37 ± 12	35 ± 15	37 ± 15	36 ± 20	0.427	0.914
Propionic acid (PA)	8.7 ± 5.3	6.5 ± 3.6	6.9 ± 4.3	6.9 ± 4.6	0.304	0.145	9.6 ± 6.5	8.9 ± 7.4	9.2 ± 6.2	11.8 ± 3.4	0.882	0.340
Iso-butyric acid (IBA)	0.60±0.39	0.61±0.45	0.62±0.45	0.55±0.57	0.461	0.861	0.62±0.55	0.60±0.55	0.66±0.55	0.67±0.52	0.621	0.660
Butyric acid (BA)	9.5 ± 6.0	6.9 ± 5.0	8.0 ± 4.9	8.0 ± 6.4	0.028 *	0.267	10.1 ± 4.5	11.2 ± 6.5	9.7 ± 4.9	10.1 ± 6.8	0.918	0.524
Valeric acid (VA)	1.04±0.59	1.00±0.53	0.91±0.53	0.81±0.66	0.339	0.894	1.32±0.99	1.29±0.71	1.28±0.95	1.35±1.18	0.725	0.808

SCFA, short-chain fatty acid; BCAA, branched-chain amino acids; mean ± SD; repeated generalized linear model; * *p* < 0.05; *p*1 = time, *p*2 = time and genotype.

## Data Availability

The data presented in this study are available on request from the corresponding author. The data are not publicly available due to the nature of the research and to enable the conduction of the intervention.

## Supplementary Materials

The following supporting information can be downloaded at: https://www.mdpi.com/article/10.3390/nu16020261/s1, Table S1: Diseases and medication usage at week 0 (n = 88). Table S2A. Correlations of plasma (n = 88) and fecal (n = 83) SCFAs with clinical characteristics, liver scores at baseline. Table S2B. Correlations of plasma BCAAs with clinical characteristics, diabetes indexes and liver scores at baseline (n = 88). Table S3. Plasma and fecal SCFA and plasma BCAA at baseline and 12 weeks. Table S4. Plasma and fecal SCFA and plasma BCAA at baseline compared with *PNPLA3* genotypes CC and GG. Table S5. Plasma and fecal SCFA and plasma BCAA at baseline compared with four study groups. Table S6. Clinical characteristics during the intervention (n = 88). Figure S1. All plasma SCFAs (a, n = 88), fecal SCFAs (b, n = 83) and plasma BCAAs (c, n = 88) at baseline (week 0) and 12 by recommended diet and average diet and PNPLA3 genotypes CC and GG (n = 83). Repeated generalized linear model; Values are presented as means ± SEM, dots/triangular dots present each participant; *p* < 0.05 in bold; *p*1 = time, *p*2 = time and genotype.
