# Supplementary material for: PNPLA3 Genotype and Dietary Fat Modify Concentrations of Plasma and Fecal Short Chain Fatty Acids and Plasma Branched-Chain Amino Acids
Source: Nutrients. 2024 Jan 16;16(2):261. doi: 10.3390/nu16020261 (PMC10819939; doi:10.3390/nu16020261)
Supplement: Supplementary file 1 [file nutrients-16-00261-s001.zip › nutrients-2723432-supplementary.pdf]

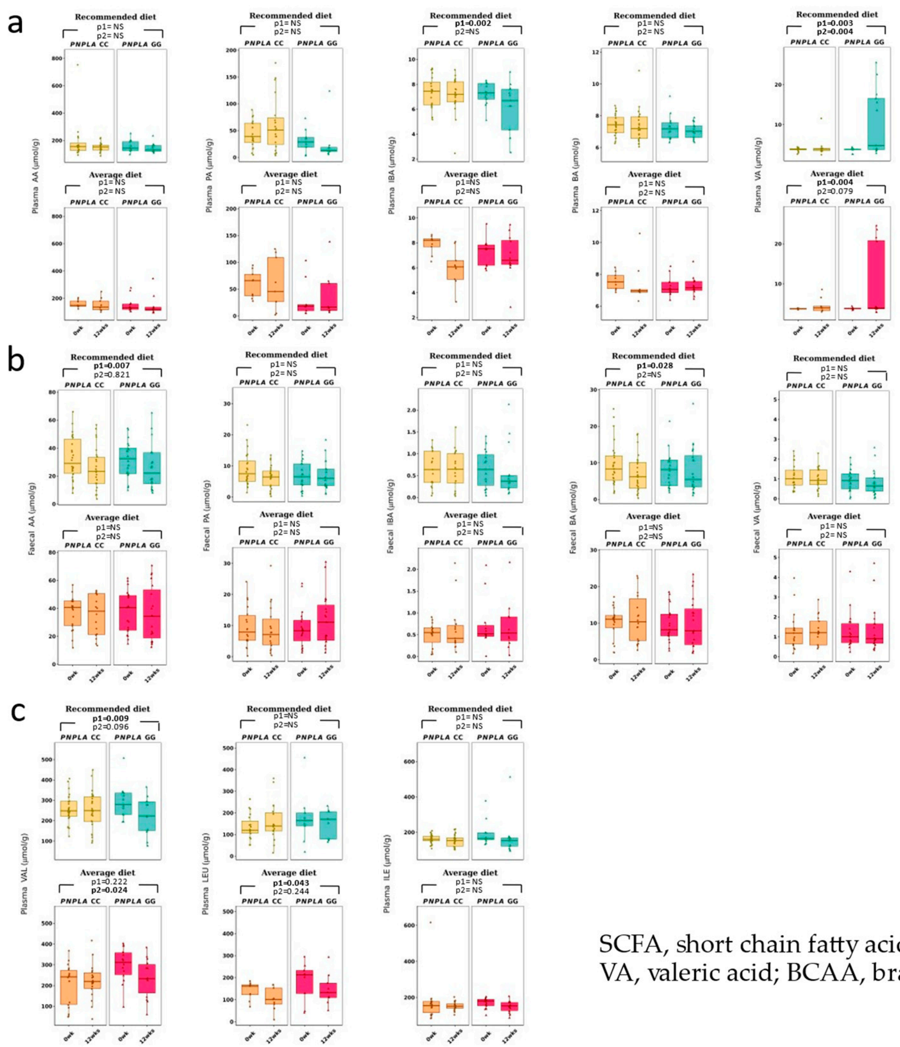

Figure S1. All plasma SCFAs (a, n = 88), fecal SCFAs (b, n = 83) and plasma BCAAs (c, n = 88) at baseline (week 0) and 12 by recommended diet and average diet and *PNPLA3* genotypes CC and GG (n = 83). Repeated generalized linear model; Values are presented as means ± SEM, dots/triangular dots present each participant; $p < 0.05$ in bold; $p1$ = time, $p2$ = time and genotype.

SCFA, short chain fatty acid; AA, acetic acid; PA, propionic acid; IBA, iso-butyric acid; BA, butyric acid; VA, valeric acid; BCAA, branched-chain amino acids; VAL, valine; LEU, leucine; ILEU, isoleucine.

Table S1. Diseases and medication usage at baseline (n = 88).

	Recommended diet		Average diet		*p*-value
	**CC**	**GG**	**CC**	**GG**	
**Total, n**	**28**	**20**	**20**	**20**	
**Diseases**					
Hypertension (%)	13 (46%)	6 (30%)	7 (35%)	12 (60%)	0.229
Coronary artery disease (%)	4 (14%)	1 (5%)	2 (10%)	1 (5%)	0.639
Cardiac insufficiency (%)	1 (4%)	-	-	1 (5%)	0.624
Stroke or transient ischemic attack (%)	1 (4%)	1 (5%)	1 (5%)	-	0.806
Cancer (%)	5 (18%)	1 (5%)	1 (5%)	-	0.116
Rheumatoid arthritis (%)	2 (7%)	-	1 (5%)	-	0.443
Inflammatory bowel disease (%)	1 (4%)	-	1 (5%)	-	0.307
Asthma (%)	2 (7%)	3 (15%)	-	1 (5%)	0.443
Hypothyroidism (%)	2 (7%)	-	1 (5%)	-	0.621
**Medications**					
Lipid lowering medication (statin / other (combined %))	16 / 1 (61%)	8 / 1 (45%)	7 / 1 (40%)	8 / 1 (45%)	0.423/0.993
Diuretics (%)	3 (11%)	-	3 (15%)	5 (25%)	0.118
Betablockers (%)	8 (29%)	3 (15%)	5 (25%)	6 (30%)	0.688
ACE / ATR (combined %)	3 / 10 (46%)	1 / 3 (20%)	3 / 3 (30%)	3 / 8 (55%)	0.730/0.131
Other antihypertensives (%)	4 (14%)	1 (5%)	3 (15%)	7 (35%)	0.078

Questionnaire-defined total (% of the group), one way ANOVA, $p < 0.05$ bolded.

Table S2A. Correlations of plasma (n = 88) and fecal (n = 83) SCFAs with clinical characteristics, liver scores at baseline.

**SFCA**	**Total SCFA**		**AA**		**PA**		**IBA**		**BA**		**VA**	
	**Plasma**	**Fecal**	**Plasma**	**Fecal**	**Plasma**	**Fecal**	**Plasma**	**Fecal**	**Plasma**	**Fecal**	**Plasma**	**Fecal**
**Cl. characteristics:**												
Waist	-0.065	0.043	-0.058	-0.005	0.061	0.102	-0.060	0.005	0.047	0.030	0.169	0.033
BMI	0.007	0.044	0.039	0.003	0.021	0.142	-0.052	-0.041	0.041	0.025	0.094	-0.003
AST	0.019	-0.126	0.054	-0.131	0.067	-0.128	0.177	0.015	0.046	-0.143	0.143	-0.065
ALT	-0.098	-0.024	-0.009	-0.036	-0.018	0.051	0.088	-0.071	-0.044	-0.058	0.078	0.024
GGT	0.023	0.106	0.091	0.109	0.103	0.080	-0.057	0.044	0.036	0.069	**0.240***	0.056
Fasting glucose	-0.126	0.144	-0.182	0.119	-0.030	0.169	-0.048	-0.043	-0.102	0.205	-0.102	0.065
Fasting insulin	-0.211	-0.063	-0.171	-0.112	0.002	0.023	-0.063	0.068	0.016	-0.024	0.009	-0.036
Total cholesterol	0.080	0.045	0.135	0.067	0.001	-0.074	0.028	-0.141	0.076	0.116	0.038	-0.092
LDL cholesterol	0.013	0.053	0.083	0.081	-0.034	-0.053	-0.001	-0.094	0.084	0.123	0.028	-0.085
HDL cholesterol	**0.302****	-0.023	**0.289****	-0.010	0.157	-0.155	0.085	-0.201	0.037	0.030	-0.032	-0.060
Triglycerides	**-0.306****	0.019	**-0.267****	0.015	-0.099	0.081	-0.088	0.065	0.029	0.021	0.056	-0.057
Hs-CRP	0.030	0.065	0.137	0.108	0.004	-0.036	0.073	-0.076	-0.002	0.057	0.163	0.048
**Diabetes indeces:**												
MATSUDA-ISI	**0.233***	0.054	**0.256***	0.094	-0.013	-0.035	0.053	-0.081	0.068	0.026	0.039	0.042
TyG	**-0.307****	0.050	**-0.283****	0.043	-0.088	0.111	-0.093	0.058	0.021	0.062	0.035	-0.034
**Liver scores:**												
FLI	-0.111	0.038	-0.079	0.008	0.042	0.109	-0.105	0.044	0.061	0.012	0.202	-0.007
HSI	-0.124	0.001	-0.016	-0.039	-0.080	0.142	-0.043	-0.041	0.008	-0.031	0.117	0.044
LFS	-0.179	0.076	-0.149	0.031	0.010	0.155	-0.021	0.032	-0.022	0.078	0.050	0.126

SCFA, short chain fatty acid; AA, acetic acid; PA, propionic acid; IBA, iso-butyric acid; BA, butyric acid; VA, valeric acid; MATSUDA-ISI, insulin sensitivity index; TyG, triglyceride glucose index (insulin resistance index), FLI, fatty liver index; HSI, hepatic steatosis index; LFS, liver fat score. Spearman, *p < 0.05 and **p < 0.005 bolded.

Table S2B. Correlations of plasma BCAAs with clinical characteristics, diabetes indeces and liver scores at baseline (n = 88).

**Plasma**	**Total BCAA**	**VAL**	**LEU**	**ILE**
**Cl. characteristics:**				
Waist	0.062	-0.044	0.088	0.123
BMI	0.217	0.027	**0.238***	0.195
AST	0.050	0.039	0.021	0.105
ALT	**0.244***	0.099	**0.295***	0.204
GGT	0.027	-0.104	0.023	-0.010
Fasting glucose	0.177	0.151	0.215	0.098
Fasting insulin	**0.331****	**0.226***	**0.336****	**0.284****
Total cholesterol	0.019	0.048	-0.004	0.100
LDL cholesterol	0.051	0.099	0.037	0.131
HDL cholesterol	-0.137	-0.152	-0.197	-0.198
Triglycerides	0.162	0.162	0.206	**0.240***
Hs-CRP	0.008	-0.052	0.045	0.049
**Diabetes indeces:**				
MATSUDA-ISI	**-0.377****	**-0.266***	**-0.397****	**-0.309****
TyG	0.189	0.187	0.299	**0.249***
**Liver scores:**				
FLI	0.153	0.021	0.187	0.205
HSI	**0.280***	0.119	**0.339****	**0.254***
LFS	**0.355****	**0.215***	**0.391****	**0.329****

BCAA, branched-chain amino acid; VAL, valine; LEU, leucine; ILE, isoleucine; BMI, body mass index; GGT, gamma-glutamyl transferase; ALT, alanine aminotransferase; AST, aspartate aminotransferase; HDL, high-density lipoprotein; LDL, low-density lipoprotein; hs-CRP, high-sensitive C-reactive protein MATSUDA-ISI, insulin sensitivity index; TyG, triglyceride glucose index (insulin resistance index); FLI, fatty liver index; HSI, hepatic steatosis index; LFS, liver fat score; Spearman, *p < 0.05 and **p < 0.005 bolded.

Table S3. Plasma and fecal SCFA and plasma BCAA at baseline (week 0) and after the intervention (week 12).

	**Recommended diet**						**Average diet**					
	**CC genotype of *PNPLA3***			**GG genotype of *PNPLA3***			**CC genotype of *PNPLA3***			**GG genotype of *PNPLA3***		
	**0**	**12**	***p*1**	**0**	**12**	***p*1**	**0**	**12**	***p*1**	**0**	**12**	***p*1**
	**28**			**20**			**20**			**20**		
**Plasma short chain fatty acids** (μmol/g)												
Total SCFA	245 ± 130	227 ± 54	0.358	232 ± 68	209 ± 85	0.068	228 ± 48	235 ± 78	0.879	219 ± 63	208 ± 61	0.964
Acetic acid (AA)	179 ± 121	145 ± 31	0.193	162 ± 63	143 ± 41	0.354	155 ± 33	167 ± 59	0.741	161 ± 60	142 ± 61	0.170
Propionic acid (PA)	48 ± 27	64 ± 52	0.675	43 ± 31	42 ± 48	0.355	52 ± 31	50 ± 44	0.199	36 ± 27	29 ± 34	0.230
Iso-butyric acid (IBA)	7.3 ± 1.4	6.6 ± 2.0	0.114	7.3 ± 1.3	5.7 ± 2.3	**0.013***	5.6 ±3.0	5.4 ± 2.5	0.839	7.3 ± 1.6	5.9 ± 2.5	0.188
Butyric acid (BA)	7.4 ± 1.0	7.3 ± 1.0	0.463	7.3 ± 1.3	5.7 ± 2.3	0.232	7.0 ± 0.7	7.2 ± 1.0	0.454	7.3 ± 0.7	6.9 ± 0.7	0.072
Valeric acid (VA)	3.7 ± 0.4	4.2 ± 1.7	0.181	3.6 ± 0.6	7.0 ± 0.6	**0.005***	3.5 ± 0.6	4.3 ± 2.1	0.127	3.8 ± 0.3	9.7 ± 8.5	**0.015***
**Plasma branched-chain amino acids** (μmol/g)												
Total BCAA	572 ± 128	581 ± 182	0.564	599 ± 202	634 ± 154	0.391	612 ± 184	532 ± 149	0.067	587 ± 182	590 ± 130	0.160
Valine (VAL)	258 ± 67	252 ± 90	0.424	266 ± 92	212 ± 95	**0.010***	196 ± 100	214 ± 89	0.476	290 ± 75	211 ± 108	**0.012***
Leucine (LEU)	136 ± 55	145 ± 85	0.676	141 ± 96	142 ± 59	0.826	141 ± 49	124 ± 71	0.068	162 ± 75	148 ± 61	0.454
Isoleucine (ILE)	160 ± 23	153 ± 33	0.236	169 ± 65	157 ± 90	0.340	164 ±112	155 ± 30.7	0.731	167 ± 26	139 ± 37	**0.009***
**Fecal short chain fatty acids** (μmol/g)	**26**			**18**			**20**			**19**		
Total SCFA	52.1 ± 25.0	39.6 ± 23.6	**0.006***	48.0 ± 21.2	42.1 ±26.2	0.690	58.5 ± 22.0	57.2 ± 26.9	0.274	57.9 ± 24.7	59.9 ± 34.8	0.802
Acetic acid (AA)	32.4 ± 15.4	25.5 ± 14.7	**0.013***	31.6 ± 13.3	26.3 ± 16.2	0.157	37.0 ± 12.3	35.3 ± 14.6	0.549	37.3 ± 15.1	36.2 ± 19.8	0.580
Propionic acid (PA)	8.7 ± 5.3	6.5 ± 3.6	0.054	6.9 ± 4.3	6.9 ± 4.6	0.786	9.6 ± 6.5	8.9 ± 7.4	0.591	9.2 ± 6.2	11.8 ± 3.4	0.415
Iso-butyric acid (IBA)	0.60 ± 0.39	0.61 ± 0.45	0.672	0.62 ± 0.45	0.55 ± 0.57	0.552	0.62 ± 0.55	0.60 ± 0.55	0.389	0.66 ± 0.55	0.67 ± 0.52	0.976
Butyric acid (BA)	9.5 ± 6.0	6.9 ± 5.0	**0.005***	8.0 ± 4.9	8.0 ± 6.4	0.528	10.1 ± 4.5	11.2 ± 6.5	0.656	9.7 ± 4.9	10.1 ± 6.8	0.643
Valeric acid (VA)	1.04 ± 0.59	1.00 ± 0.53	0.390	0.91 ± 0.53	0.81 ± 0.66	0.546	1.32 ± 0.99	1.29 ± 0.71	0.649	1.28 ± 0.95	1.35 ± 1.18	0.943

SCFA, short chain fatty acid; AA, acetic acid; PA, propionic acid; IBA, iso-butyric acid; BA, butyric acid; VA, valeric acid; BCAA, branched-chain amino acids; VAL, valine; LEU, leucine; ILE, isoleucine; mean ± SD, repeated generalized linear model, *$p < 0.05$ bolded, $p1$ = time.

Table S4. Plasma and fecal SCFA and plasma BCAA at baseline compared with *PNPLA3* genotypes CC and GG.

	CC genotype of *PNPLA3*	GG genotype of *PNPLA3*	*p*-value
**Plasma short chain fatty acids** (μmol/g)	**n = 48**	**n = 40**	
Total SCFA	243 ± 105	225 ± 65	0.372
Acetic acid (AA)	169 ± 95	162 ± 61	0.662
Propionic acid (PA)	50 ± 28	40 ± 29	0.125
Iso-butyric acid (IBA)	6.6 ± 2.3	7.3 ± 1.5	0.106
Butyric acid (BA)	7.3 ± 0.9	7.3 ± 1.0	0.745
Valeric acid (VA)	3.6 ± 0.5	3.7 ± 0.4	0.592
**Plasma branched-chain amino acids** (μmol/g)			
Total BCAA	585 ± 148	617 ± 177	0.416
Valine (VAL)	231 ± 88	278 ±84	**0.014***
Leucine (LEU)	137 ± 52	152 ± 86	0.376
Isoleucine (ILE)	162 ± 74	168 ± 49	0.648
**Fecal short chain fatty acids** (μmol/g)	**n = 46**	**n = 37**	
Total SCFA	54.7 ± 23.7	52.8 ± 23.2	0.718
Acetic acid (AA)	34.3 ± 14.3	34.4 ± 14.3	0.969
Propionic acid (PA)	9.1 ± 5.6	8.0 ± 5.4	0.386
Iso-butyric acid (IBA)	0.6 ± 0.5	0.6 ± 0.5	0.435
Butyric acid (BA)	9.7 ± 5.4	8.8 ± 4.9	0.742
Valeric acid (VA)	1.2 ± 0.8	1.1 ± 0.8	0.730

SCFA, short chain fatty acid; AA, acetic acid; PA, propionic acid; IBA, iso-butyric acid; BA, butyric acid; VA, valeric acid; BCAA, branched-chain amino acids; VAL, valine; LEU, leucine; ILE, isoleucine; mean ± SD, one way ANOVA, Bonferroni, *$p < 0.05$ bolded.

Table S5. Plasma and fecal SCFA and plasma BCAA at baseline compared with four study groups.

	RD and CC genotype of *PNPLA3*	RD and GG genotype of *PNPLA3*	AD and CC genotype of *PNPLA3*	AD and GG genotype of *PNPLA3*	*p*-value
**Plasma short chain fatty acids** (μmol/g)	**n = 28**	**n = 20**	**n = 20**	**n = 20**	
Total SCFA	245 ± 130	232 ± 68	228 ± 48	219 ± 63	0.594
Acetic acid (AA)	179 ± 121	162 ± 63	155 ± 33	161 ± 60	0.760
Propionic acid (PA)	48 ± 27	43 ± 31	52 ± 31	36 ± 27	0.378
Iso-butyric acid (IBA)	7.3 ± 1.4	7.3 ± 1.3	5.6 ±3.0	7.3 ± 1.6	**0.012***
Butyric acid (BA)	7.4 ± 1.0	7.3 ± 1.3	7.0 ± 0.7	7.3 ± 0.7	0.519
Valeric acid (VA)	3.7 ± 0.4	3.6 ± 0.6	3.5 ± 0.6	3.8 ± 0.3	0.139
**Plasma branched-chain amino acids** (μmol/g)					
Total BCAA	572 ± 128	599 ± 202	612 ± 184	587 ± 182	0.679
Valine (VAL)	258 ± 67	266 ± 92	196 ± 100	290 ± 75	**0.005***
Leucine (LEU)	136 ± 55	141 ± 96	141 ± 49	162 ± 75	0.652
Isoleucine (ILE)	160 ± 23	169 ± 65	164 ±112	167 ± 26	0.965
**Fecal short chain fatty acids** (μmol/g)	**n = 26**	**n = 18**	**n = 20**	**n = 19**	
Total SCFA	52.1 ± 25.0	48.0 ± 21.2	58.5 ± 22.0	57.9 ± 24.7	0.453
Acetic acid (AA)	32.4 ± 15.4	31.6 ± 13.3	37.0 ± 12.3	37.3 ± 15.1	0.457
Propionic acid (PA)	8.7 ± 5.3	6.9 ± 4.3	9.6 ± 6.5	9.2 ± 6.2	0.442
Iso-butyric acid (IBA)	0.60 ± 0.39	0.62 ± 0.45	0.62 ± 0.55	0.66 ± 0.55	0.631
Butyric acid (BA)	9.5 ± 6.0	8.0 ± 4.9	10.1 ± 4.5	9.7 ± 4.9	0.979
Valeric acid (VA)	1.04 ± 0.59	0.91 ± 0.53	1.32 ± 0.99	1.28 ± 0.95	0.285

SCFA, short chain fatty acid; AA, acetic acid; PA, propionic acid; IBA, iso-butyric acid; BA, butyric acid; VA, valeric acid; BCAA, branched-chain amino acids; VAL, valine; LEU, leucine; ILE, isoleucine; mean ± SD, one way ANOVA, Bonferroni, *$p < 0.05$ bolded.

Table S6. Clinical characteristics during the intervention (n=88).

	Recommended diet				Average diet					
	CC genotype of *PNPLA3*		GG genotype of *PNPLA3*		CC genotype of *PNPLA3*		GG genotype of *PNPLA3*		*p1*	*p2*
**Total, n**	**28**		**20**		**20**		**20**			
**Study week**	**0**	**12**	**0**	**12**	**0**	**12**	**0**	**12**		
BMI (kg/m^2)	27.8 ± 2.5	27.7 ± 2.5	25.8 ± 2.0	25.7 ± 2.1	28.1 ± 2.4	28.1 ± 2.6	26.4 ± 2.3	26.3 ± 2.3	0.105	0.903
Waist (cm)	101.5 ± 8.7	100.9 ± 9.0	94.6 ± 7.6	94.3 ± 8.0	104.4 ± 8.0	104.2 ±	95.8 ± 7.5	95.2 ± 7.3	0.71	0.925
GGT (U/L)	32.8 ± 19.3	33.5 ± 19.8	20.1 ± 5.6	18.8 ± 5.6	32.8 ± 21.1	32.8 ± 20.0	26.1 ± 9.9	26.3 ± 13.2	0.259	0.488
ALT (U/L)	26.3 ± 13.3	27.4 ± 11.9	23.5 ± 12.9	22.0 ± 10.3	27.7 ± 9.1	28.3 ± 8.9	27.7 ± 10.6	27.8 ± 10.1	0.940	0.625
AST (U/L)	28.2 ± 7.4	29.2 ± 6.2	27.0 ± 6.9	27.6 ± 6.6	27.6 ± 5.0	28.1 ± 8.4	27.0 ± 7.5	27.9 ± 5.9	0.135	0.880
Albumin (g/dL)	38.3 ± 2.8	38.6 ± 2.3	38.0 ± 2.4	38.4 ± 2.8	38.9 ± 2.2	38.7 ± 2.3	38.5 ± 3.2	38.6 ± 3.0	0.352	0.758
Total cholesterol (mmol/L)	4.21 ± 0.97	4.27 ± 1.04	4.51 ± 0.80	4.37 ± 0.75	4.66 ± 0.96	4.98 ± 0.97	4.58 ± 1.01	4.87 ± 0.98	**0.014***	**0.011***
HDL cholesterol (mmol/L)	1.47 ± 0.54	1.46 ± 0.35	1.49 ± 0.37	1.46 ± 0.36	1.29 ± 0.25	1.39 ± 0.29	1.41 ± 0.37	1.46 ± 0.34	**0.030***	**0.009***
LDL cholesterol (mmol/L)	2.53 ± 0.77	2.48 ± 0.83	2.84 ± 0.78	2.59 ± 0.73	3.04 ± 0.86	3.26 ± 0.85	2.93 ± 0.89	3.14 ± 0.95	0.488	**0.002***
Triglycerides (mmol/L)	0.97 ± 0.37	1.03 ± 0.44	1.21 ± 0.79	2.0 ± 0.89	1.28 ± 0.42	1.32 ± 0.37	1.12 ± 0.49	1.06 ± 0.35	0.719	0.778
Fasting glucose (mmol/L)	5.71 ± 0.45	5.70 ± 0.43	5.58 ± 0.35	5.58 ± 0.45	5.78 ± 0.35	5.86 ± 0.56	5.83 ± 0.41	5.76 ± 0.49	0.770	0.889
120min glucose (mmol/L)	6.23 ± 1.46	5.80 ± 1.53	5.81 ± 1.46	5.84 ± 1.43	6.31 ± 1.67	5.98 ± 2.04	5.96 ± 1.16	6.22 ± 1.46	0.244	0.254
Fasting insulin (mU/L)	9.1 ± 5.4	9.1 ± 5.7	7.4 ± 3.5	6.8 ± 0.8	14.0 ± 7.6	13.5 ± 8.3	9.1 ± 5.4	10.2 ± 9.0	0.225	0.696
120min insulin (mU/L)	63.2 ± 58.6	63.1 ± 64.6	40.9 ± 27.2	43.1 ± 27.3	59.8 ± 40.6	57.8 ± 40.4	52.1 ± 46.3	80.1 ± 95.8	0.408	0.243
Hs-CRP (mg/L)	1.01 ± 1.04	1.15 ± 1.39	0.71 ± 0.31	1.07 ± 0.94	1.65 ± 1.45	1.49 ± 1.39	1.02 ± 0.87	1.23 ± 1.07	0.336	0.710

BMI, body mass index; GGT, gamma-glutamyl transferase; ALT, alanine aminotransferase; AST, aspartate aminotransferase; HDL, high-density lipoprotein; LDL, low-density lipoprotein; hs-CRP, high-sensitive C-reactive protein; mean ± SD, repeated generalized linear model, *$p < 0.05$ bolded, $p1$ = time, $p2$ = time and genotype.
